# Effectiveness of Psychoeducation on Burden among Family Caregivers of Adults with Schizophrenia: A Systematic Review and Meta-Analysis

**DOI:** 10.1155/2023/2167096

**Published:** 2023-09-19

**Authors:** Akunna Jane Okafor, Mark Monahan

**Affiliations:** School of Nursing and Midwifery, Trinity College Dublin, Dublin, Ireland

## Abstract

Caring for relatives living with schizophrenia could lead to caregivers' burden. It is believed that lack of information and understanding about schizophrenia and lack of skills to cope effectively while caring for their adult relatives largely contribute to the burden they experience. The burden is assessed using assessment scales. This review aims to assess the effectiveness of psychoeducation in alleviating the burden experienced by family caregivers of adults living with schizophrenia and to identify essential factors that facilitate positive outcomes. Five databases (ASSIA, CINAHL, Embase, MEDLINE EBSCO, and PsycINFO) were systematically searched using combinations of the following key terms: “family caregivers,” “schizophrenia,” “burden,” “psychoeducation,” and “adults.” Meta-analysis of included studies was conducted using RevMan 5.4. Five RCTs with 320 family caregivers were included in the review. Overall, none of the studies showed a low risk of bias. The evidence suggests that face-to-face group psychoeducation reduced family caregivers' burden when measured across different time points: one-week postintervention (mean difference −3.87 and Cl −6.06 to −1.70), six months (MD −8.76 and Cl −12.38 to −5.13), and twelve months (MD −7.38 and Cl −9.85 to −4.91). Measurements immediately after the intervention, one month, and three months postintervention when reported narratively also showed a reduction in family caregivers' burden. Face-to-face group psychoeducation provided for family caregivers effectively alleviates the burden they experience. Factors such as program content and teaching methods facilitated positive outcomes. It is recommended that psychoeducation should be integrated as a routine intervention for family caregivers.

## 1. Introduction

The effective promotion of recovery-oriented care for adults living with a diagnosis of schizophrenia is considered to include not just providing interventions for the person but also providing adequate support to family and caregivers[1–3].

Schizophrenia is a severe mental health difficulty that affects 7 per 1,000 of the adult population [4]. While its incidence is low, its prevalence is high due to the enduring nature of the symptoms associated with this mental health difficulty [5]. Even with effective pharmacological interventions for managing positive symptoms, adults living with schizophrenia may still experience adverse symptoms. This may limit their ability to be financially stable and care for themselves independently [6].

Family caregivers play an essential role in supporting the care and recovery of adults living with schizophrenia [7]. Studies suggest that 90% of adults experiencing schizophrenia live with family members when discharged from hospitals [2, 7]; hence, they depend on the assistance and involvement of family and caregivers in managing symptoms and providing support, including emotional and financial support [8].

Caring for adults living with schizophrenia has been linked to increased family caregivers' burden [9–14]. Due to the intensity and diversity of caregiving, family caregivers may experience burdens as either physical, emotional, and financial or as a combination of these factors [2, 12, 15–17]. The Global Burden of Disease Report [18] highlighted that family caregivers often ignore their own emotional, physical, and mental health while providing care for their relatives; hence, resulting in severe stress, depression, and anxiety [19]. The longer the caregiving role, the greater burden the family caregivers' experience [7, 17, 19–21].

Lippi [8] identified that family caregivers experience caregiving burdens due to lack of information and understanding of schizophrenia as well as lack of skills to cope with the symptoms the person may be experiencing [8].

Psychoeducation is recommended as an intervention to provide support and information to family caregivers (National Institute for Health and Care Excellence (NICE) 2014).

However, despite the significant burden experienced by family caregivers of adult relatives living with schizophrenia, few primary studies have specifically investigated the impact of psychoeducation on family caregivers' burden without including their adult relatives in the studies. This is possibly because family caregivers' needs are not considered as important as the needs of individuals experiencing schizophrenia [9–13, 22–26].

Few studies have conducted systematic reviews in exploring caregiving-related outcomes for family caregivers [26–31]; however, none have explicitly focused on the concept of burden experienced by family caregivers. Furthermore, no systematic review of randomised controlled trial studies carried out on the effectiveness of psychoeducational programs on the burden experienced by family caregivers of adults living with schizophrenia was identified in the Prospero register for systematic review.

This systematic review aims to determine the effectiveness of psychoeducational programs on the burden experienced by family caregivers of adults living with schizophrenia.

The objectives were to investigate the effectiveness of face-to-face psychoeducational programs on the burden experienced by family caregivers of adults living with schizophrenia using the Family Burden Interview Schedule and to identify essential factors that facilitate positive outcomes.

## 2. Methods

This systematic review was conducted following the Preferred Reporting Items for Systematic Reviews and Meta-Analyses (PRISMA) statement checklist [32]. There was no review protocol for this study.

### 2.1. Inclusion/Exclusion Criteria

The inclusion and exclusion criteria used in this review are outlined using PICOS (population, intervention, comparators, outcomes, and study design) [33].

Studies were eligible and included in this review only where they reported on the following terms.

- Population: This means family caregivers of adults living with schizophrenia, where the person given the diagnosis is an adult aged 18 years and above. They may be experiencing first episode of schizophrenia or enduring schizophrenia. For this systematic review, family caregivers are defined as family members related either biologically, such as parents, children, siblings, and grandparents; or nonbiologically, such as spouses, and friends [34]. Relatives must be adults of 18 years and above.
- Intervention: This means studies evaluating the use of face-to-face psychoeducational programs delivered to family caregivers. Studies were included if most of the psychoeducation sessions were delivered to family caregivers (family caregivers focused). Studies were also included if the content of the psychoeducational program aimed to improve the family caregivers' experience of care and reduce their burden. To qualify as a psychoeducational intervention program, the program's design must include an educational component that impacts knowledge and provides information on schizophrenia and its management.
- Comparator/control: This include family caregivers who received routine care.
- Outcome: This means caregiving-related outcome (caregivers' burden).
- Study type: Randomised controlled trial (RCT) studies were included.

Studies were excluded from the review if they did not meet the inclusion criteria.

### 2.2. Primary Outcome

The primary outcome of interest of the review is family caregivers' burden measured pre- and postintervention and compared to the control group, using a tool that measured family burdens. As identified earlier, several tools were devised to explore family burden. In this instance, research using the Family Burden Interview Schedule (FBIS) [16] was selected over other family caregivers' burden assessment tools because

- The Family Burden Interview Schedule (FBIS) specifically assesses burden experienced by family caregivers of adults living with schizophrenia.
- The FBIS has proven validity and reliability with Cronbach's *α* of 0.90.
- The FBIS measures both objective and subjective burdens.
- The FBIS has been used in both community and inpatient settings.
- The FBIS has a broad international base of studies and is the most widely used tool in research and literature.
- The Zarit Caregiver Burden Interview [15] has not been assessed for internal consistency (Cronbach' *α*). In addition, the tool was initially developed to assess the burden experienced by family caregivers of older adults living with dementia.
- The Perceived Family Burden Scale (PFBS) [35] and the Family Burden Scale (FBS) [36] have not had wide use, and it was not possible to extract comparable data from these tools.

### 2.3. Search and Selection Strategy

In May 2021, electronic searches of five databases, Applied Social Sciences Index and Abstracts (ASSIA), Cumulative Index to Nursing and Allied Health Literature (CINAHL), Embase, MEDLINE EBSCO, and PsycINFO, were conducted from the date of inception to May 2021 by the first author. Firstly, scoping reviews were searched using databases thesauri in MEDLINE EBSCO, CINAHL, Embase, ASSIA, and PsycINFO. The keywords were selected based on the elements of PICOS (population, interventions, comparator/control, outcome, and study design). The keywords include “family caregivers,” “schizophrenia,” “burden,” “psychoeducation,” and “adults.” In order not to unintentionally exclude relevant articles, the comparator/control and study design elements were not included in the keywords.

Following that, keywords contained in the title, abstract, and subject terms used to describe the articles retrieved during the scoping searches were analysed and used in the main search. A search string was developed to maximise the validity of the review. Each concept (population, intervention, and outcome) was searched individually, using the keywords and the MeSH terms combined with the Boolean operator “OR.” In the end, the different concepts were combined using the Boolean operator “AND.”

This search strategy was initially developed for MEDLINE EBSCO and then adapted for all other searched databases using the keywords and database-specific subject headings. No limitations were applied. This was to ensure that all relevant articles were captured.

Grey literature online search was conducted on Cochrane Library (https://www.cochranelibrary.com/); Lenus, the Irish Health Repository (https://www.lenus.ie/hse/); and Open Grey (https://opengrey.eu/). Web-based review of European Fedaration of Families of People with Mental Illness (EUFAMI) was conducted. Conference proceedings of relevant conferences were also examined.

Searches of the reference lists of the articles selected for inclusion in the review were conducted. In addition, the reference list for the Cochrane review of family intervention for schizophrenia [37] was also reviewed for relevant papers.

Table 1 is a search strategy result using MEDLINE EBSCO database retrieved on 19 May 2021.

### 2.4. Study Selection

Covidence (https://www.covidence.org/) was used in managing the review's screening and selection process. Citations retrieved from the search were uploaded to Covidence. The selection process was conducted in 2 stages. Articles were initially screened on the title and abstract. For stage 2, potential eligible articles were screened for full text. Any article that did not meet the inclusion criteria of this review, articles not written in English, or articles not empirically researched were excluded. The initial screening and selection process was conducted by the first author and then checked by the second author.

## 3. Results of the Search and Selection Process

The electronic database search yielded a total of 143 citations. MEDLINE EBSCO yielded 35 articles, CINAHL: 18, Embase: 20, ASSIA: 40, and PsycINFO: 30 articles. All the articles were saved in EndNote and uploaded to Covidence for screening. The search on Cochrane Library (https://www.cochranelibrary.com/) yielded ten citations. Lenus, the Irish Health Repository (https://www.lenus.ie/hse/), yielded three citations and Open Grey (https://opengrey.eu/) yielded a further three articles; none were relevant to the review. Furthermore, reference lists of selected articles yielded two citations. Only one was included in the review [26]. Two citations were retrieved from the reference list for the Cochrane review of family intervention for schizophrenia [37]. A search of conference proceedings did not yield any citation relevant to the review. In total, 163 citations were screened using Covidence.

Of the 163, 104 citations were removed, as they were identified as duplicates, leaving 59 citations. Following the title and abstract screening, 41 citations were excluded as they were not relevant for the review as psychoeducation was not used as an intervention. Full-text papers of the remaining 18 citations were obtained and reviewed for eligibility. Of these 18 citations, one was excluded because the full text was not written in English [38]. Six studies were removed as they did not meet the inclusion criteria; intervention was not family caregivers-focused as caregivers did not have their psychoeducational sessions without their relatives living with schizophrenia present [39–44]. Two of the studies were not randomised controlled trial studies [45, 46]. Four studies were excluded as they did not measure their outcome data with Family Burden Interview Schedule [11–13, 25]. Five studies were identified to be suitable for inclusion in this review [9, 22–24, 26].  Table 2 presents the excluded studies.

**Figure 1:**
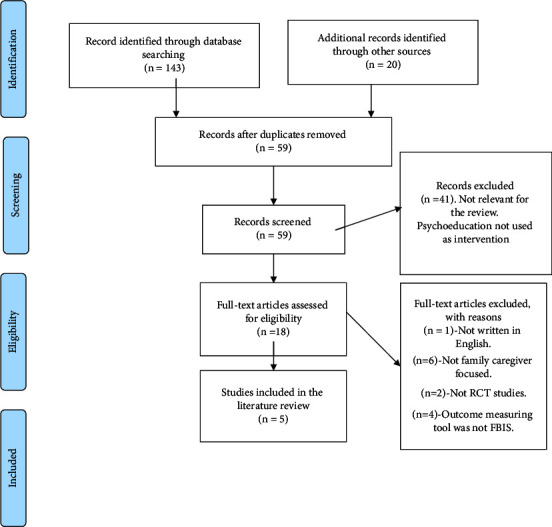
PRISMA flow chart. Source: from Liberati et al. 2009, the PRISMA statement for reporting systematic reviews and meta-analyses of studies that evaluate health care interventions: explanation and elaboration. *Journal of Clinical Epidemiology ***62** 1–34.

Figure 1 provides a visual representation of the review's search and selection strategy using the PRISMA framework.

### 3.1. Data Extraction

A data extraction form based on a template from the authors' affiliated institution was adapted to extract data from all included studies. The form was piloted in one study before being used in the rest of the included studies. Data extracted included the study design, setting, participants, inclusion and exclusion criteria, description of the intervention, comparisons, any reported data related to the reviews' outcome (continuous data), and participants' sociodemographic characteristics. As one of the objectives of this review is to evaluate the essential factors that facilitate reported outcomes, relevant data on duration, format, and teaching methods of psychoeducational programs were also extracted and presented in narrative format. Authors of published articles were contacted to retrieve relevant information about their study that was either not reported or unclear from the published article. The first author independently extracted the data from the selected studies. The second author verified the extracted data.

### 3.2. Quality Assessment

The quality of all included articles was assessed using the Cochrane risk of bias tool. Cochrane risk of bias was used because it promotes transparency in the systematic review by assessing the risk that may affect the study's validity rather than assigning score to different items in a scale [33]. The quality assessment was performed independently by the two authors. Differences were resolved through discussion.

### 3.3. Narrative Result of the Quality Assessments of the Included Studies

#### 3.3.1. Random Sequence Generation (Selection Bias)

Cheng and Chan [22] and Fallahi Khoshknab et al. [26] had a low risk of bias on random sequence generation due to using drawing lots and block randomisation, respectively. Risk of bias was unclear in the study by Chien et al. [24], Chien and Wong [23], and Koolaee and Etemadi [9] due to a lack of insufficient information to make a “low” or “high” risk judgment.

#### 3.3.2. Allocation Concealment (Selection Bias)

Fallahi Khoshknab et al. [26] had a low risk of allocation concealment bias as opaque sealed envelopes were used. The risk of bias was unclear in the remaining four studies; the method of concealment was not described in sufficient detail to allow “low risk” or “high risk” judgment.

#### 3.3.3. Blinding of Participants and Personnel (Performance Bias)

The study by Chien and Wong [23] was judged as having a low risk of bias as they reported that one researcher was blinded to the participant's allocation. The other four studies' risk of bias was assessed as unclear due to insufficient information to permit judgment of “low risk” or “high risk.”

#### 3.3.4. Blinding of Outcome Assessment (Detection Bias)

The risk of bias on this criterion was assessed as a low risk in the study by Chien and Wong [23]. This is because one of the researchers administered the pretest and post-test was blinded. Cheng and Chan [22], Chien et al. [24], Koolaee and Etemadi [9], and Fallahi Khoshknab et al. [26] did not provide enough information to judge the detection bias as “low risk” or “high risk”; therefore was assessed as unclear.

#### 3.3.5. Incomplete Outcome Data (Attrition Bias)

All five studies were assessed and judged as low risk in this criterion because all studies reported a low attrition rate. However, only one study (Chien et al. [24]) performed an intention-to-treat analysis of the results.

#### 3.3.6. Selective Reporting (Reporting Bias)

All studies were assessed and judged as having a low risk of bias on selective reporting as all prespecified outcomes were reported.

#### 3.3.7. Other Bias

**Figure 2:**
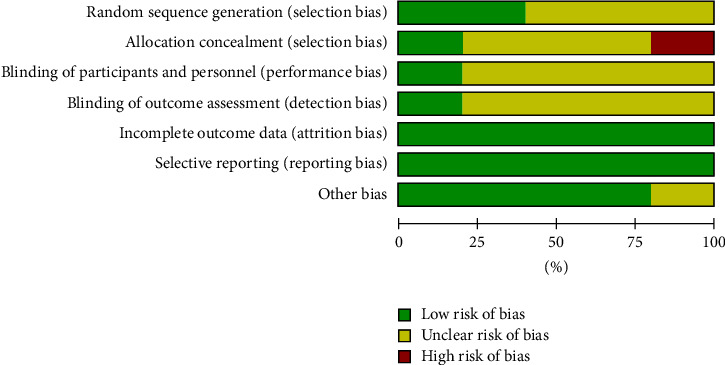
Risk of bias graph using cochrane risk of bias tool [33].

**Figure 3:**
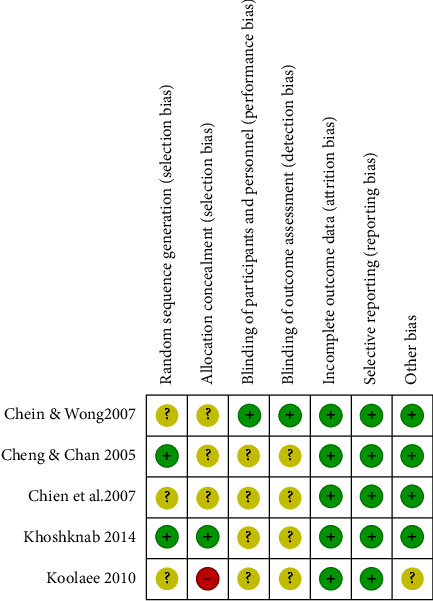
Risk of bias summary using cochrane risk of bias tool [33]. 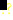 Yellow indicates unclear. 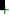 Green indicates low risk. 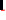 Red indicates high risk.

Of all the five studies, only the study by Koolaee and Etemadi [9] was assessed and judged as unclear. This is because of insufficient information to assess whether any significant risk of bias exists. The remaining four studies were assessed as low risk of bias to other sources of bias. Figures 2 and 3 illustrate the overall quality assessment of the included studies using the risk of bias graph and risk of bias summary.

Figures 2 and 3 illustrate the overall quality assessment of the included studies using the risk of bias graph and the risk of bias summary, respectively.

## 4. Data Synthesis

Data synthesis in this review was achieved through meta-analysis, as there were similarities among the included studies.

- The randomised controlled trial studies included in this systematic review have similar populations
- The included studies compare similar interventions and comparators
- The included studies report similar outcomes either as primary or secondary outcomes
- The findings of the included randomised control trial studies report similar results; that is, the studies determined that one intervention is better than another or there was no difference between the interventions

The following time points grouped the outcome data: immediately after the intervention, one week after intervention (follow-up), six months, and 12 months after intervention. Those that could not be grouped were reported narratively. The common essential factors which may facilitate a positive outcome were analysed narratively.

The data analysis was carried out using the review manager (RevMan 5.4).

For each time point group, for example, studies grouped for one week after intervention, the mean, standard deviation, and a total number of participants of both the intervention and the control groups, as reported in the studies, were inputted into RevMan 5.4.

The mean and standard deviation of studies were inputted in RevMan 5.4 because the outcome measure for this review was the continuous data (burden of caregivers of adults living with schizophrenia). The mean difference (MD) with a 95 percent confidence interval (CI) was calculated. The mean difference was used because all included studies for this review measured their outcome with the same measurement tool (Family Burden Interview Schedule). After that, forest graphs were plotted for each of the grouped time points. Heterogeneity was assessed in this systematic review. The statistical test used to assess heterogeneity is the *I* squared (*I*^2^) statistic, which was automatically calculated in RevMan during meta-analysis.

### 4.1. Description of Included Studies

A total of 320 participants were involved in this review. All five included studies reported the primary outcome for this review, which is the burden experienced by family caregivers of adults living with schizophrenia. The psychoeducational programs delivered in all five studies were focused on family caregivers. In 4 studies, Cheng and Chan [22], Chien et al. [24], Koolaee and Etemadi [9], and Fallahi Khoshknab et al. [26], relatives living with schizophrenia did not attend any sessions. In one study (Chen and Wong [23]) family caregivers' adult relatives living with schizophrenia attended only 6 out of 18 psychoeducational sessions, which were focused on education about schizophrenia, its symptoms, management, and the effects of medications.

All five studies measured family caregivers' burden using Family Burden Interview Schedule. All five studies measured their reported outcome using continuous data. They all reported the primary outcome for this review, which is the burden experienced by caregivers of adults living with schizophrenia. However, they measured their reported outcome at different time points. Of the five studies, only two studies, Cheng and Chan [22] and Fallahi Khoshknab et al. [26], reported their measured outcomes immediately after the intervention. Chien et al. [24] and Chien and Wong [23] reported their measured outcomes one week after the intervention. At time point one month, only one study, Fallahi Khoshknab et al. [26], reported their measured outcome. In a similar vein, only one study, Koolaee and Etemadi [9], reported their measured outcome after three months postintervention. Two of the included studies reported their measured outcome six months postintervention [9, 24]. At 12 months postintervention, two studies, Chien et al. [24] and Chien and Wong [23], reported their measured outcome.

Two studies had two arms of intervention; Chien et al. [24] compared psychoeducation *n* = 33, mutual support *n* = 32, and routine care *n* = 31 and Koolaee and Etemadi [9] compared psychoeducation *n* = 19, behavioural group therapy *n* = 18, and routine care = 18. However, for this review, only data related to psychoeducation intervention and routine care data were extracted.

Table 3 illustrates the summary characteristics of the included studies.

## 5. Meta-Analysis

### 5.1. Main Outcome-Caregivers' Burden

In total, five studies were analysed. One study was reported in *t* value and *p* value, and the remaining four studies were pooled. All studies indicated a decrease in caregivers' burden after the delivery of psychoeducational programs, when measured with the Family Burden Interview Schedule (FBIS) at different time points.

### 5.2. Effect of Intervention Immediately after the Intervention

Two studies, Cheng and Chan [22] and Fallahi Khoshknab et al. [26], assessed the effect of psychoeducation immediately after intervention. Reporting narratively on the result of Cheng and Chan [22], the psychoeducation group (*n* = 32) had a pretest reading of 18.78 and posttest reading of 11.06 compared to the control group (*n* = 32) that had a pretest reading of 17.03 and posttest reading of 16.28. The reported *t* value was 5.25 and *p* value was 000. This result indicates that family caregivers received psychoeducation experienced less burden immediately after the intervention. The reported p value is less than 0.01(<0.01), indicating that the result is statistically significant using an alpha cut-off level of *p* = 0.05.

Fallahi Khoshknab et al. [26] reported a mean score of 27.87 and a standard deviation score of ±2.9 for the intervention group (*n* = 36) compared to the mean score of 37.82 and standard deviation of ±2.78 for the control group (*n* = 35). The reported *p* value was <0.01. This indicates that family caregivers who received psychoeducation had better outcomes than those who received routine care. Based on the *p* value that is <0.01, using an alpha cut-off level of *p*=0.05, the result is statistically significant.

### 5.3. Effect of Intervention One Week after Intervention

**Figure 4:**
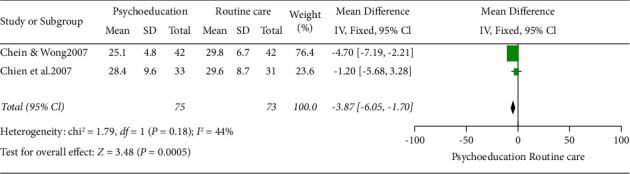
Fixed effect model forest plot one week after intervention.

Two studies were included in this meta-analysis (Chien and Wong [23] and Chien et al. [24]) with a total number of 148 participants. The overall result showed a decrease in the burden experienced by family caregivers in the psychoeducation group compared to the control group after one week of delivering psychoeducation (Figure 4). The result was statistically significant (2 RCTs, *n* = 148, MD: 3.87, Cl: −6.06 to −1.70, *I*^2^: 44%). Among the two studies included, Chen and Wong [23] contributed more to the information with the weight of 76.4%. This could be because a greater number of participants were included in the study. Fixed effect model was used as the heterogeneity (*I*^2^) was less than 50 percent Figure 4.

### 5.4. Effect of Intervention after One Month

Reporting narratively on the results from Fallahi Khoshknab et al. [26], the mean score for the intervention group was 21.3 and standard deviation was ±2.78 compared to a mean score of 37.3 and standard deviation of ±2.81 in the control group with reported *p* value<0.01. This showed a lower score of burden in the intervention group compared to the control group. The result was statistically significant.

### 5.5. Effect of Intervention after Three Months

Reporting narratively on the results from Koolaee and Etemadi [9], the mean score for the intervention group was 25.84 and standard deviation of ±9.10 with nineteen participants compared to the mean score of 45.11 and a standard deviation of ±9.47 in the control group with eighteen participants, with a reported *p* value <0.01. The result showed a lower score of family caregivers' burden in the intervention group compared to the control group. The result was statistically significant.

### 5.6. Effect of Intervention after Six Months

Two studies were grouped in this meta-analysis (Chien et al. [24] and Koolaee and Etemadi [9]) with 101 participants based on analysis six months postintervention. The overall result showed that psychoeducation had a significant positive effect in reducing burden six months postintervention when compared with routine care.

**Figure 5:**
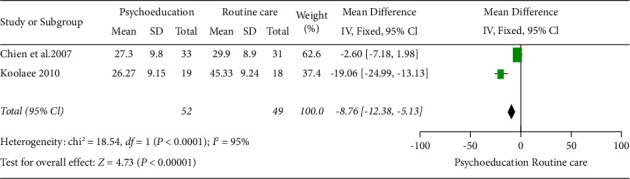
Fixed effect model forest plot 6 months after the intervention.

The result was statistically significant (2 RCTs, *n* = 101, MD: −8.76, Cl: −12.38 to −5.13, and *I*^2^ = 95%) (Figure 5).

**Figure 6:**
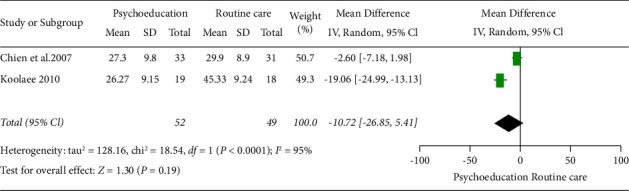
Random effect model forest plot 6 months after intervention.

Of note, the heterogeneity of the two studies was high, (95%). Hence, random effect model meta-analysis was also completed in RevMan 5.4, which also revealed a high heterogeneity of *I*^2^ = 95% (Figure 6) for the random effect model forest plot.

### 5.7. Effect of Intervention after Twelve Months

**Figure 7:**
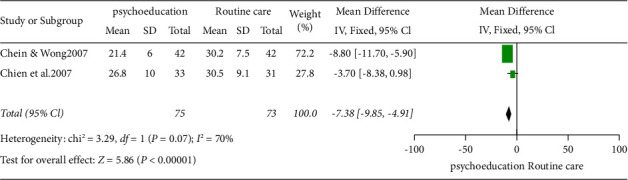
Fixed effect forest plot model twelve months after intervention.

Two studies, Chien and Wong [23] and Chien et al. [24], with 148 participants assessed the effect of psychoeducation on family caregivers' burden 12 months after delivery. The result of the meta-analysis indicated a decrease in family caregivers' burden after 12 months compared to those that received routine care. The result is statistically significant (2 RCTs, *n* = 148, MD: −7.38, Cl: −9.85 to −4.91, and *I*^2^ = 70%) (Figure 7).

### 5.8. Common Essential Intervention Factors Which Facilitated Positive Outcome

The duration of the psychoeducational interventions reported by the five studies included studies ranged from four sessions to eighteen sessions; that is, duration was either brief or long. Regarding format, all included studies used a group format. Multimodal teaching methods were used in the five studies, such as group discussion, problem-solving skills, and teaching. Chien et al. [24], Chien and Wong [23], and Koolaee and Etemadi [9] used some strategies to facilitate the participants' attendance, such as advanced reminders, repeating sessions on weekends, regular telephone follow-up, and running sessions at convenient locations. Mental health professionals facilitated psychoeducational programs in all five studies.  Table 4 summaries the common essential factors which facilitated positive outcomes in terms of their duration (brief (four weeks) vs. long (≥ ten weeks) programs), delivery format, teaching methods used, and factors/strategies that facilitated the attendance of participants.

## 6. Discussion

The overall finding of this meta-analysis showed that psychoeducational programs aimed at family caregivers of adults living with schizophrenia were effective in alleviating their burden compared to routine care. This finding is similar to the findings of other systematic reviews [26–30]. Despite the methodological difference in their respective reviews, their findings demonstrated burden reduction in the family caregivers that received psychoeducational programs compared to the family caregivers that received routine care. However, this systematic review differs, as it represents a meta-analysis of 320 participants from five RCTs explicitly focused on family caregivers' burden measured using the Family Burden Interview Schedule (FBIS).

Of the five studies included in this systematic review, three of the included studies [22–24] reported that family caregivers experienced moderate burden at baseline. Two studies, Koolaee and Etemadi [9] and Fallahi Khoshknab et al. [26], reported that at baseline, family caregivers experienced severe burden. Fallahi Khoshknab et al. [26] reported a total burden score of over 40 on a scale of 0–48 of the Family Burden Interview Schedule (FBIS), indicating severe burden; the higher the score of the total burden on the FBIS, the more severe the burden experienced.

This review identified the burden experienced by family caregivers at different time points after receiving psychoeducation; four of the studies, [9, 23, 24], and [26], showed a lowered mean score, while one study [22] showed a lowered post-test score after the delivery of psychoeducational program (see Section 5).

Two studies [22, 26] measured their outcome immediately after delivering their psychoeducational program. In two studies, Chien et al. [24] and Chien and Wong [23] measured the burden outcome one week postintervention. The burden was also measured at one month [26], three months [9], six months [9, 24], and one year [23, 24] after providing psychoeducation to family caregivers. The finding from this systematic review, aligns with those of earlier studies that identified positive effects of psychoeducation on the burden experienced by family caregivers of adults living with schizophrenia when measured at different time points. Sharif et al. [10] measured the burden experienced by 70 family caregivers one month after they had received psychoeducation. In Greece, Palli et al. [11] used a waiting-list control study design to identify reduced levels of burden in family caregivers immediately after and one year postreceiving a psychoeducational program.

Furthermore, Thimmajja and Lazarus Rathinasamy [13] identified reduction in family caregivers' burden after one month and three months they had engaged in a psychoeducational program. This indicates that psychoeducational programs aimed at family caregivers of adults living with schizophrenia are effective immediately after it is delivered; effectiveness can be sustained up to one year after. Family caregivers were likely practicing the skills gained during the intervention, resulting in reduced burden. Cheng and Chan [22] and Fallahi Khoshknab et al. [26] identified that the effectiveness of psychoeducation provided for family caregivers gets better over time. However, ongoing psychoeducation may be required to maintain the learned skills [34, 47].

The duration of the psychoeducational programs reported by the five studies included in this systematic review ranged from four sessions [26] to eighteen sessions [9, 22–24]. According to Zhao et al. [48], any psychoeducational program with less than ten sessions is considered a brief psychoeducational program. This shows that short and long psychoeducational programs can effectively reduce the burden experienced by family caregivers. This finding supports the finding of an earlier study by Worakul et al. [49] who aimed to evaluate the effectiveness of brief psychoeducation on the knowledge and attitude of family caregivers. They provided a 1-day intense psychoeducational program to 91 family caregivers of adults living with schizophrenia.

Their findings showed improved knowledge of schizophrenia and positive attitude. The effectiveness of brief psychoeducation was further intensified by Thimmajja and Lazarus Rathinasamy's [13] RCT prepost control group design study. They noticed that the mean burden score of family caregivers reduced from 82.37 to 49.13 after one month and 40.86 three after months postpsychoeducation program. This indicated that brief psychoeducation was effective in the reduction of family caregivers' perceived burden.

Furthermore, Sota et al. [50], in their dose-response design, identified that the positive effect of psychoeducational programs depended not only on the duration of psychoeducational program provided but on the content of the program and teaching methods.

The contents of psychoeducational programs in all the five studies included in this review reported education about schizophrenia and its management as their cardinal element. Their psychoeducational programs content tended to be delivered in a modular design, as the topics were spread out over their various duration. According to Hughes and Quinn [51], programs designed in modular format are more effective than traditional teaching design in the teaching and learning process. Programs designed in modular format enable adult learners to learn at their pace. It also provides an opportunity for learners to practice; this encourages motivation and promotes active participation. Cheng and Chan [22] and Gutiérrez-Maldonado and Caqueo-Urízar [25] suggested that the participants' cultural backgrounds should be considered when planning and designing psychoeducational programs. This is because mental health difficulties are understood and interpreted differently across different ethnicity and culture [27]. This is supported by Hughes and Quinn [51] who recommended that the design of any educational program and its information content should suit the diverse needs of adult learners. The common topics/contents learned by family caregivers as reported by the five included studies in this review include the aetiology, symptoms, and management of schizophrenia. In addition, problem-solving skills, communication skills, and information on available local resources were also delivered.

The learning experience was beneficial in improving family caregivers' skills to communicate with their relatives and deal with caregiving challenges more effectively [22]. This could explain the improvement identified in some categories of the FBIS, such as disruption of routine family activities, disruption of family leisure, and disruption of family interaction [22, 26]. This supports Ewers et al. [52], Tabeleão et al. [12], and Thimmajja and Lazarus Rathinasamy [13]; they highlighted that a better understanding of the nature of schizophrenia, its symptoms, and its effect on their relatives' behaviour would likely result in a change of attitude. Family caregivers may have a new perspective on the caregiving experience and change their cognitive appraisal [22]. In addition, Thimmajja and Lazarus Rathinasamy [13] suggested that information provided in the psychoeducational programs would enable family caregivers to recognise their relatives' behavioural deficits as negative symptoms of schizophrenia instead of referring to their relatives as being lazy; as a result, experience fewer burden [22]. It could be said that family caregivers experienced reduced financial burdens due to increased use of services as a result of increased knowledge of available resources [9]. This shows that the content of psychoeducational programs could be said to be one of the essential factors that facilitated positive outcomes in the family caregivers in the psychoeducational group.

Other essential factors that may have facilitated the positive outcome in this review include the use of a face-to-face group format, the various teaching methods, and the engagement strategies used. All the five included studies in this review delivered their psychoeducational program through a face-to-face group format. This enabled the use of teaching methods such as group discussion in all the included studies [9, 22–24] and Fallahi Khoshknab et al. [26] and role-play [22, 24].

The use of a group discussion teaching method in a face-to face group format could have given family caregivers in the psychoeducation group the opportunity to share and learn from others' personal experiences. This supports Copper et al. [53] and Bengo [54]; they highlighted that group activities promote active learning by allowing adult learners to contribute their different ideas and experiences to the group. This provided social support for the family caregivers; as they realised that they are not alone and that other family caregivers face similar issues [22, 24]. This is supported by Sin and Norman's [27] mixed method systematic review. Their qualitative analysis indicates that family caregivers that received their psychoeducational program in a group format reported experiencing peer support and a reduced sense of isolation. In addition, the group discussion in the group format helped to normalise their caring experience and boosted their self-efficacy; as a result, it reduced the caregiving burden they were experiencing.

The use of strategies such as reminding participants a day before scheduled sessions, repeating sessions, and running sessions at convenient community settings to encourage attendance could have also contributed to the positive outcome of the psychoeducational programs, as low dropout rates were reported [22–24].

This systematic review identified that psychoeducation could be delivered in inpatient and outpatient settings. However, only two of the included studies in this review [22, 26] provided their psychoeducational program in inpatient settings. This is similar to the findings of earlier studies by Nilsen et al. [55], Petrakis and Laxton [34], and Nolan and Petrakis [56]. In their respective qualitative studies, Nilsen et al. [55], Petrakis and Laxton [34], and Nolan and Petrakis [56] identified that fewer psychoeducational programs for family caregivers are provided in the inpatient settings. This is attributed to insufficient time by staff working in the inpatient setting. Hence, the emphasis is on more inpatient psychoeducational programs for family caregivers; especially those whose adult relatives are experiencing first episode of schizophrenia [56].

Furthermore, this review shows that members of the multidisciplinary team can conduct psychoeducational programs. It also showed that mental health nurses are well-positioned to facilitate psychoeducation. This is because mental health nurses have regular contact with family caregivers and, most times, comprehend their needs [22]. In this review, four of the included studies [22–24, 26] reported that mental health nurses conducted their psychoeducational programs. However, Higgins et al. [57] suggested involving family caregivers as cofacilitators. This will give the family caregivers a chance to interact and learn from those that have lived the experience [24].

This systematic review identified that most of the family caregivers that participated in the included studies were female gender (*n* = 212), 66.25 percent. This indicates the female gender is mostly the primary family caregivers of their adult relatives living with schizophrenia; hence, it could be said that they experience more caregiving-related burdens than males. This is in line with the findings of [58, 18, 25]. To this effect, Gutiérrez-Maldonado and Caqueo-Urízar [25] suggested that gender and family roles should be considered while developing psychoeducational programs for family caregivers of adults living with schizophrenia.

Despite the positive outcome of psychoeducation on burden identified in this review, face-to-face psychoeducational programs are yet to be implemented regularly in the practice setting for family caregivers due to factors that can impede its implementation. These include transportation and time constraints on the part of the family caregivers [11, 25, 26]. The findings of this systematic review support this. Four of the included studies reported drop out of family caregivers during their respective studies [9, 23, 24, 26]. Only two of the studies, Chien et al. [24] and Koolaee and Etemadi [9], reported reasons for drop out (Table 3). The reasons for these dropouts were that family members had to travel long distances to attend face-to-face psychoeducational programs. The other reason was that family caregivers were not available to attend due to time clashing with other commitments.

In addition, Mottaghipour and Tabatabaee [59] highlighted that shame and stigma due to family caregivers' relatives' mental health difficulties could hinder them from participating in face-to-face psychoeducation. Furthermore, Chien et al. [24] and Coulthard et al. [60] identified that psychoeducation could be expensive to implement as facilitators are paid for facilitating the psychoeducational programs. Therefore, the cost could be a barrier to the successful implementation of psychoeducation in mental health services.

This indicates that lack of easy accessibility to the location of psychoeducational programs, time constraints, shame, stigma, and cost could pose barriers in the implementation of family psychoeducation in the practice setting.

In as much as this review identified that psychoeducational programs are effective in the reduction of burden experienced by family caregivers of adults living with schizophrenia; caution should be taken to interpret the overall result.

### 6.1. Strengths and limitations

The robustness of the review process is one of this systematic review's strengths. There is obvious evidence of a robust search strategy and thorough literature. In addition, the quality appraisal was conducted independently by two researchers. However, it is essential to note that this study has some limitations. Only studies published in English language were included, potentially introducing publication bias. One reviewer independently extracted the data. This could have introduced data extraction errors. All the included studies were conducted in mental health settings in the Asian population, where healthcare practices are more likely to differ from those in other countries, therefore limiting the generalisability of findings. Hence, there is a need for more studies on family caregivers' burdens to be conducted globally.

The methodological quality of included studies limits this review's findings, as none of the studies were judged as having a low risk of bias overall. Publication bias could have been introduced in this review by excluding studies that did not measure family caregivers' burden with Family Burden Interview Schedule. Finally, the high heterogeneity identified in the review may have limited the external validity of the findings.

## 7. Conclusion/Implications for Practice

Although there are limitations in this review, the evidence indicates that psychoeducation has positive effects on the burden experienced by family caregivers at all the time points assessed. It is recommended that assessing the level of caregiving burden experienced should be added to the routine assessment. This review has identified the effectiveness of even brief psychoeducation; as such, it should be included as a routine intervention for family caregivers in acute inpatient mental health settings.

## Figures and Tables

**Table 1:** Medline search strategy and results.

#	Query	Limiters/expander	Results
S16	S11 AND S12 AND S13 AND S14 AND S15	Search mode: Boolean/phrase	35
S15	S5 OR S10	Search mode: Boolean/phrase	6,054,920
S14	S4 OR S9	Search mode: Boolean/phrase	282,813
S13	S3 OR S8	Search mode: Boolean/phrase	1,235,724
S12	S2 OR S7	Search mode: Boolean/phrase	2,350,020
S11	S1 OR S6	Search mode: Boolean/phrase	160,225
S10	(“MH Adult^*∗*^”) OR (MH “young adult”) OR (MH “adult children^*∗*^”)	Search mode: Boolean/phrase	6,034,941
S9	(“MH caregiver burden”) OR (MH “burden^*∗*^”) OR (MH “exhaustion^*∗*^”) OR (MH “burnout^*∗*^”)	Search mode: Boolean/phrase	272,813
S8	(MH “schizophrenia”) OR (MH “schizophrenia disorganized”) OR (MH “schizophrenia paranoid”) OR (MH “schizophrenia catatonic”) (MH “schizophrenia spectrum”) OR (MH “mental Disorders+”)	Search mode: Boolean/phrase	1,228,120
S7	(MH “caregivers”) OR (MH “caregiving”) OR (MH “family intervention”) OR (MH “family”) OR (MH “family relations”) OR (MH “family conflict”) OR (MH “nuclear family+”) OR (MH “parents+”)	Search mode: Boolean/phrase	2, 300,035
S6	(MH “psychoeducation”) OR (MH “education”+) OR (MH “teaching”) (MH “models, educational”)	Search mode: Boolean/phrase	90,810
S5	TI (adult^*∗*^” OR “young adult^*∗*^“) OR AB (adult^*∗*^“) OR “young adult^*∗*^”)	Search mode: Boolean/phrase	6,034941
S4	TI (“caregiver burden” OR “burden^*∗*^” OR “exhaustion^*∗*^” OR “burnout”) OR AB (“caregiver burden” OR “burden^*∗*^” OR “exhaustion^*∗*^” OR “burnout”)	Search mode: Boolean/phrase	262,813
S3	TI (“schizophrenia^*∗*^” OR “psychosis^*∗*^” OR “psychotic illness^*∗*^” OR “schizophrenic disorders^*∗*^” OR “mental disorder^*∗*^” OR AB (“schizophrenia^*∗*^” OR “psychosis^*∗*^” OR “psychotic illness^*∗*^” OR “schizophrenic disorders^*∗*^” OR “mental disorder^*∗*^”	Search mode: Boolean/phrase	148,138
S2	TI (“family caregiver^*∗*^” OR “caregiver^*∗*^” OR “caregiver^*∗*^” OR “informal caregiver^*∗*^” OR “unpaid family caregiver^*∗*^” OR “informal carer^*∗*^” OR “carer^*∗*^” OR “home nursing^*∗*^” OR “relative care^*∗*^” “couples”^*∗*^ OR “daughter”^*∗*^ OR “family”^*∗*^ OR “father”^*∗*^ OR “friend”^*∗*^ OR “husband”^*∗*^ OR “marital”^*∗*^ OR “mother”^*∗*^ or “multifamily”^*∗*^ OR “neighbour^*∗*^” OR “next of kin^*∗*^” OR “friend^*∗*^” OR “niece^*∗*^” OR “nephew^*∗*^”OR “parent^*∗*^” OR “partner^*∗*^” OR “relative^*∗*^” OR sibling^*∗*^” OR “significant other^*∗*^” OR “spouse^*∗*^” OR “son^*∗*^” OR “step relationship^*∗*^” OR “wife^*∗*^” OR AB (“family caregiver^*∗*^” OR caregiver^*∗*^” “informal caregiver^*∗*^” OR “unpaid family caregiver^*∗*^” OR “informal carer^*∗*^” OR “carer^*∗*^” OR “home nursing^*∗*^” OR “relative care^*∗*^” “couples”^*∗*^ OR “daughter”^*∗*^ OR “family”^*∗*^ OR “father”^*∗*^ OR “friend”^*∗*^ OR “husband”^*∗*^ OR “marital”^*∗*^ OR “mother”^*∗*^ OR “multifamily”^*∗*^ OR “neighbour^*∗*^” OR “next of kin^*∗*^” OR “friend^*∗*^” OR “niece^*∗*^” OR “nephew^*∗*^” OR “parent^*∗*^” OR “partner^*∗*^” OR “relative^*∗*^” OR “sibling^*∗*^” OR “significant other^*∗*^” OR “spouse^*∗*^” OR “son^*∗*^” OR “step relationship^*∗*^” OR “wife^*∗*^”	Search mode: Boolean/phrase	2,224,035
S1	TI (“psychoeducation^*∗*^” OR “psycho-education^*∗*^” OR “psychoeducational program^*∗*^” OR “workshop^*∗*^” OR “training program^*∗*^” OR “educational activity^*∗*^” OR “face-to face” OR “group session^*∗*^” OR “group intervention^*∗*^” OR “education^*∗*^” OR “instruction^*∗*^” OR teaching^*∗*^” OR AB (“psychoeducation^*∗*^” OR “psycho-education”^*∗*^ “psychoeducational program^*∗*^” “workshop”^*∗*^ OR “training program^*∗*^” OR “educational activity^*∗*^” “face-to-face” OR “group session^*∗*^” OR “group intervention^*∗*^” OR “education^*∗*^” OR “instruction^*∗*^” OR “teaching^*∗*^”)	Search mode: Boolean/phrase	80,300

**Table 2:** Studies excluded from the review.

Study	Reason for exclusion
Kane et al. [45]	Non-RCT
Birchwood et al. [46]	Non-RCT
[38]	Not written in English
Das et al. [39]	Intervention not carer-focused (no carer, only sessions)
Gutiérrez-Maldonado and Caqueo-Urízar [25]	Did not measure burden with FBIS
Kulhara et al. [40]	Intervention not carer-focused (no carer, only sessions)
Gonzalez-Blanch et al. [41]	Intervention not carer-focused (no carer, only sessions)
Fiorillo et al. [42]	Intervention not carer-focused (no carer, only sessions)
Palli et al. [11]	Did not measure burden with FBIS
Bulut et al. [44]	Intervention not carer-focused (no carer, only sessions)
Purba and Bukit [43]	Intervention not carer-focused (no carer, only sessions)
Tabeleão et al. [12]	Did not measure burden with FBIS
Thimmajja and Lazarus Rathinasamy [13]	Did not measure burden with FBIS

**Table 3:** Summary characteristics of included studies for systematic review (methodological table).

Study number	Author, year, and country	Population	Intervention, duration, and format	Comparator	Study design	Tools	Follow-up	Drop out/reason	Gender of caregiver	Mean age of caregivers (years)
1	Cheng and Chan [22], China	64 Intervention *n* = 32 control = 32	Face-to-face psychoeducation. Group. 10 sessions × 2 hours	Routine care (RC)	RCT	Family Burden Interview Schedule (FBIS)	No follow	None Reason: not provided	F-40 M-24	Not reported

2	Chien et al. [24] China	64 Intervention *n* = 33 control *n* = 31	Psychoeducation. Group. 12 sessions × 2 hours	RC	RCT	FBIS	6.12, 18 months	3 from intervention group 2 from control group Reason: long distance	F-19 M-45	45

3	Chien and Wong [23], China	84 Intervention *n* = 42 control *n* = 42	Psychoeducation. Group. 18 sessions × 2 hours	RC	RCT	FBIS	One week, 12 months	3 from intervention group 4 from control group Reason: not provided	F-56 M-28	41

4	Fallahi Khoshknab et al. [26], Iran	71 Intervention *n* = 36 control *n* = 35	Psychoeducation. Group. 4 sessions × 2 hours weekly	RC	RCT	FBIS	One month	5 from intervention group during follow-up Reason: not provided	F- 60 M-11	54

5	Koolaee and Etemadi [9], Iran	37 Intervention *n* = 19 control *n* = 18	Psychoeducation. Group. 12 sessions × 2 hours Weekly	RC	RCT	FBIS	3, 6 months	2 from intervention group 2 from control group Reason: unavailable to attend	F-37 mothers M-0	55

**Table 4:** Common essential factors which facilitated positive outcome.

Study	Format	Psychoeducation delivered by/setting	Factors that facilitated attendance	Duration of psychoeducation	Teaching methods	Contents of psychoeducational programs
[22]	Group face-to-face	Mental health nurse inpatients	Advanced reminders Repeated sessions per weekend	Long program	Teaching and group discussion	Aetiology, symptoms and management of schizophrenia problem-solving skills, communication skills, and information on available local resources

Chien et al. [24]	Group face-to-face	Mental health nurse outpatients	Regular telephone follow-up	Long program	Group discussion and teaching	Aetiology, symptoms and management of schizophrenia Problem-solving skills, and communication skills

[23]	Group face-to-face	Mental health nurse outpatients	Running sessions at a convenient community setting	Long program	Group discussion, workshop, and teaching	Aetiology, symptoms, and management of schizophrenia Problem-solving skills, and communication skills

[26]	Group face-to-face	Mental health nurse. Inpatients	Not reported	Brief program	Group discussion, provision of handout, and teaching	Aetiology and symptoms and management of schizophrenia Information on available local resources

[9]	Group face-to-face	Mental health nurse. Outpatients	Not reported	Long program	Group discussion, provision of handout, and teaching	Aetiology and symptoms and management of schizophrenia Problem-solving skills, communication skills, and information on available local resources

## Data Availability

The data that support the findings of this study are available from the corresponding author upon reasonable request.
